# Genomics dataset on unclassified published organism (patent US 7547531)

**DOI:** 10.1016/j.dib.2016.09.046

**Published:** 2016-10-05

**Authors:** Mohammad Mahfuz Ali Khan Shawan, Md. Ashraful Hasan, Md. Mozammel Hossain, Md. Mahmudul Hasan, Afroza Parvin, Salina Akter, Kazi Rasel Uddin, Subrata Banik, Mahbubul Morshed, Md. Nazibur Rahman, S.M. Badier Rahman

**Affiliations:** Department of Biochemistry and Molecular Biology, Jahangirnagar University, Savar, Dhaka 1342, Bangladesh

**Keywords:** Genomics dataset, patent US 7547531, NCBI BioSample database, QR code, GC content, Cleavage code, Hierarchical classification, Taxonomic position

## Abstract

Nucleotide (DNA) sequence analysis provides important clues regarding the characteristics and taxonomic position of an organism. With the intention that, DNA sequence analysis is very crucial to learn about hierarchical *classification of that particular organism. This dataset* (patent US 7547531) is chosen to simplify all the complex raw data buried in undisclosed DNA sequences which help to open doors for new collaborations. In this data, a total of 48 unidentified DNA sequences from patent US 7547531 were selected and their complete sequences were retrieved from NCBI BioSample database. Quick response (QR) code of those DNA sequences was constructed by DNA BarID tool. QR code is useful for the identification and comparison of isolates with other organisms. AT/GC content of the DNA sequences was determined using ENDMEMO GC Content Calculator, which indicates their stability at different temperature. The highest GC content was observed in GP445188 (62.5%) which was followed by GP445198 (61.8%) and GP445189 (59.44%), while lowest was in GP445178 (24.39%). In addition, New England BioLabs (NEB) database was used to identify cleavage code indicating the 5, 3 and blunt end and enzyme code indicating the methylation site of the DNA sequences was also shown. These data will be helpful for the construction of the organisms’ hierarchical classification, determination of their phylogenetic and taxonomic position and revelation of their molecular characteristics.

**Specifications Table**

**Table:** 

Study area	Biological Sciences
More definite study area	Genomics, Microbiology, Bioinformatics
Data types	Table, figure, graph and QR Code
How data was obtained	Through NCBI BioSample database
Data format	Raw and analyzed
Experimental factors	Dataset obtained through bioinformatics tool
Experimental features	Only disclosed genome sequences were used
Data source location	Department of Biochemistry and Molecular Biology, Jahangirnagar University, Savar, Dhaka-1342, Bangladesh.
Data accessibility	Data available within this article and via the NCBI repository http://www.ncbi.nlm.nih.gov/nuccore/?term=patent+US+7547531

**Value of the data**

• Data regarding AT and GC percentage of the DNA sequences would give idea about their stability at different temperatures.
• The QR code would be useful for the identification*,* qualitative, quantitative analysis of the isolates and for their comparison with other organisms.
• These data give information about exact position of restriction sites to create blunt and sticky ends and also give an idea about the sites where cleavage is being affected by methylation.

## 1 Data

This paper contains data on quick response (QR) codes, guanine and cytosine (GC) content, analyzed DNA sequences and microorganisms having similar regions of 48 nucleotide sequences of unclassified disclosed microorganism from patent US 7547531. All the sequences of unidentified microorganisms disclosed from the patent US 7547531 were downloaded in FASTA format via NCBI nuccore database. These retrieved nucleotide sequences were utilized to generate QR codes, calculate GC content along with GC plot, determine number of cleavage code (blunt end cut, 5′ and 3′ sticky ends extension) and identify number of enzyme code (cleavage affected by CpG and other methylation).

## 2 Experimental design, materials and methods

At the beginning, a total of 48 nucleotide sequences (GP445164, GP445165, GP445166, GP445167, GP445168, GP445169, GP445170, GP445171, GP445172, GP445173, GP445174, GP445175, GP445176, GP445177, GP445178, GP445179, GP445180, GP445181, GP445182, GP445183, GP445184, GP445185, GP445186, GP445187, GP445188, GP445189, GP445190, GP445191, GP445192, GP445193, GP445194, GP445195, GP445196, GP445197, GP445198, GP445199, GP445200, GP445201, GP445202, GP445203, GP445204, GP445205, GP445206, GP445207, GP445208, GP445209, GP445210 and GP445211) of unclassified published microorganism from patent US 7547531 were retrieved from most trustworthy biological databases namely NCBI (National Center for Biotechnology Information) via Nucleotide DNA database (http://www.ncbi.nlm.nih.gov/nuccore/?term=patent+US+7547531) and saved in FASTA format [1], [2]. The QR code for each of the nucleotide sequence was determined by using DNA BarID tool (http://www.neeri.res.in/DNA_BarID/DNA_BarID.htm) [3] (Supplementary Table 1). GC content as well as GC plot of each nucleotide sequence was analyzed by ENDMEMO DNA/RNA GC Content Calculator (http://www.endmemo.com/bio/gc.php). GC content was determined as percentage of guanine (G) and cytosine (C) nucleotides in a given sequence (Supplementary Table 2), while GC plot was the blueprint of G and C nucleotide allotment in a given sequence illustrated through graphical image (Supplementary Fig. 1). Within the GC plot, middle blue line show average GC percentage, while upper and lower red lines indicate maximum and minimum percentage of GC allotment respectively [4], [5], [6], [7], [8]. The analysis of large non-overlapping open reading frames within a given nucleotide sequence was determined by using NEBcutter V2.0 tool (http://nc2.neb.com/NEBcutter2/) [9]. For each sequence, this tool determines possible number of cleavage in the form of blunt end cut and 5′ and 3′ sticky ends extension, while the identified number of enzyme code provide precise clue about cleavage affected by CpG and other types of methylation (Supplementary Table 2 and Supplementary Fig. 2). Furthermore, New England BioLabs (NEB) database determined the A (adenine) and T (thymine) as well as GC percentages in nucleotide sequence [10]. After that, nucleotide blast (https://blast.ncbi.nlm.nih.gov/Blast.cgi) was done for each of the disclosed unidentified nucleotide sequence to identify the regions of similarity between biological sequences [11], [12].

## References

[1] Shawan M.M.A.K., Hossain M.M., Hasan M.M., Parvin A., Akter S., Uddin K.R., Banik S., Morshed M., Hasan M.A., Rahman M.N., Rahman S.M.B. (2015). *In silico* characterization and investigation of putative promoter motifs in *Ebolavirus* genome. J. Glob. Biosci..

[2] Shawan M.M.A.K., Hossain M.M., Hasan M.A., Hasan M.M., Parvin A., Akter S., Uddin K.R., Banik S., Morshed M., Rahman M.N., Rahman S.M.B. (2015). Design and prediction of potential RNAi (siRNA) molecules for 3׳UTR PTGS of different strains of zika virus: a computational approach. Nat. Sci..

[3] Rekadwad B.N., Khobragade C.N. (2016). Digital data for quick response (QR) codes of alkalophilic *Bacillus pumilus* to identify and to compare bacilli isolated from Lonar Crator Lake, India. Data Brief.

[4] Rekadwad B.N., Khobragade C.N. (2016). Digital data for Quick Response (QR) codes of thermophiles to identify and compare the bacterial species isolated from Unkeshwar hot springs (India). Data Brief.

[5] Rekadwad B.N., Khobragade C.N. (2016). Data on true tRNA diversity among uncultured and bacterial strains. Data Brief.

[6] Rekadwad B.N., Khobragade C.N. (2016). Digital data of quality control strains under general deposit at Microbial Culture Collection (MCC), NCCS, Pune, India: a bioinformatics approach. Data Brief.

[7] Rekadwad B.N., Khobragade C.N. (2016). Bioinformatics data supporting revelatory diversity of cultivable thermophiles isolated and identified from two terrestrial hot springs, Unkeshwar, India. Data Brief.

[8] Rekadwad B.N., Khobragade C.N. (2016). Determination of GC content of *Thermotoga maritima*, *Thermotoga neapolitana* and *Thermotoga thermarum* strains: a GC dataset for higher level hierarchical classification. Data Brief.

[9] Vincze T., Posfai J., Roberts R.J. (2003). NEBcutter: a program to cleave DNA with restriction enzymes. Nucleic Acids Res..

[10] J. Domenech-Casal, Gene Hunting: una secuencia contextualizada de indagación alrededor de la expresión génica, la investigación in silico y la ética en la comunicación biomedical, Revista Eureka sobre Enseñanza y Divulgación de las Ciencias, 13 (2016) 342–358.

[11] Shawan M.M.A.K., Mahmud H.A., Gope P.S., Salauddin N.M., Rahman M.H., Ahmed M.A., Nafiz T.N., Imran K.M., Rahman M.N., Rahman S.M.B. (2016). *Campylobacter jejuni* ATCC 700819: an in silico approach to identify and categorize probable drug targets by subtractive genome analysis. J. Pure Appl. Microbiol..

[12] Shawan M.M.A.K., Mahmud H.A., Hasan M.M., Parvin A., Rahman M.N., Rahman S.M.B. (2014). *In Silico* modeling and immunoinformatics probing disclose the epitope based peptide vaccine against zika virus envelope glycoprotein. Indian J. Pharm. Biol. Res..

