# Supplementary material for: Genomics dataset on unclassified published organism (patent US 7547531)
Source: Data Brief. 2016 Oct 5;9:602–5. doi: 10.1016/j.dib.2016.09.046 (PMC5066183; doi:10.1016/j.dib.2016.09.046)
Supplement: Supplementary file 1 — Supplementary material [file mmc1.docx]

**Conflict of interest**

The authors do not declare any competing interest.
